# Small Renal Masses without Gross Fat: What Is the Role of Contrast-Enhanced MDCT?

**DOI:** 10.3390/diagnostics12020553

**Published:** 2022-02-21

**Authors:** Gerta Repeckaite, Kristina Zviniene, Justina Jankauskiene, Algidas Basevicius, Daimantas Milonas

**Affiliations:** 1 Department of Radiology, Medical Academy, Lithuanian University of Health Sciences, 44307 Kaunas, Lithuania; kristina.zviniene@lsmuni.lt (K.Z.); justina.jankauskiene@lsmuni.lt (J.J.); algidas.basevicius@lsmuni.lt (A.B.); 2 Department of Urology, Medical Academy, Lithuanian University of Health Sciences, 44307 Kaunas, Lithuania; daimantas.milonas@lsmuni.lt

**Keywords:** multi-detector computed tomography, small renal neoplasms, incidental findings

## Abstract

Increased detection of small renal masses (SRMs) has encouraged research for non-invasive diagnostic tools capable of adequately differentiating malignant vs. benign SRMs and the type of the tumour. Multi-detector computed tomography (MDCT) has been suggested as an alternative to intervention, therefore, it is important to determine both the capabilities and limitations of MDCT for SRM evaluation. In our study, two abdominal radiologists retrospectively blindly assessed MDCT scan images of 98 patients with incidentally detected lipid-poor SRMs that did not present as definitely aggressive lesions on CT. Radiological conclusions were compared to histopathological findings of materials obtained during surgery that were assumed as the gold standard. The probability (odds ratio (OR)) in regression analyses, sensitivity (SE), and specificity (SP) of predetermined SRM characteristics were calculated. Correct differentiation between malignant vs. benign SRMs was detected in 70.4% of cases, with more accurate identification of malignant (73%) in comparison to benign (65.7%) lesions. The radiological conclusions of SRM type matched histopathological findings in 56.1%. Central scarring (OR 10.6, *p* = 0.001), diameter of lesion (OR 2.4, *p* = 0.003), and homogeneous accumulation of contrast medium (OR 3.4, *p* = 0.03) significantly influenced the accuracy of malignant diagnosis. SE and SP of these parameters varied from 20.6% to 91.3% and 22.9% to 74.3%, respectively. In conclusion, MDCT is able to correctly differentiate malignant versus uncharacteristic benign SRMs in more than 2/3 of cases. However, frequency of the correct histopathological SRM type MDCT identification remains low.

## 1. Introduction

As various non-invasive imaging modalities, mainly ultrasound (US) and computed tomography (CT), become increasingly more accessible and utilized, incidental findings, such as renal lesions, are detected more frequently [1,2,3]. The majority of incidental renal findings are typical benign cysts, which are observed in up to 40% of adult patients and require no treatment [4]. Solid tumours, however, pose a clinical challenge; some of them are benign or non-aggressive and do not require treatment, while others must be treated early to ensure a good prognosis [5,6]. Renal cell carcinomas (RCCs) usually fall into the latter category, and contribute to 3–5% of oncological diseases worldwide, which means that with more than 50% of RCCs being diagnosed incidentally, optimal differentiation is imperative [7,8]. Typically, a biopsy is performed to determine the malignancy and type of the renal tumour, while imaging studies evaluate lesion invasion, spread, and related complications [9]. However, intervention is not always possible, and has its own complications. Biopsy results may be inconclusive, especially in smaller tumours, and surgical treatment may be unnecessary due to the indolence of the mass [10,11,12]. As the number of incidentally detected renal lesions increases, less invasive and cost-effective methods to differentiate between benign and malignant tumours are required. Due to its availability, reasonable cost, and accurate assessment of tumour morphology, location, and size, multi-detector CT (MDCT) is being researched as a modality of choice for solid renal mass evaluation.

Many of incidentally detected renal lesions are less than 4 cm in diameter, commonly described as small renal masses (SRMs), and may be benign (typically angiomyolipomas (AMLs) or oncocytomas) or malignant, the most common of which are the RCCs [4,8]. In MDCT, the malignancy and type of a solid renal mass can be determined, or at least suspected, by assessing various aspects of the tumour: homogeneity and intensity of enhancement, scarring, calcification, macroscopic fat, and size and contours of the lesion, etc. However, certain limitations apply; the mass has to be at least 1 cm in diameter for its enhancement to be accurately measurable, attenuation changes of 10–20 Hounsfield units (HU) are inconclusive, and the smaller the mass, the more difficult it is to observe calcification, necrotic, or adipose tissue [13]. Furthermore, no single radiological characteristic of a lesion is definitive when determining not only the type, but also the malignancy of the lesion.

In cases that the lesion has macroscopic fat or shows signs of aggressive growth, MDCT diagnosis of malignancy is relatively easy. Unfortunately, SRM appearance is not always typical. For example, central scarring is indicative of both benign and malignant lesions: oncocytoma, clear cell carcinoma (CCC), papillary RCCs, and epithelioid AMLs [14,15]; and AMLs may be lipid-poor and visually similar to RCCs, or large, solid, heterogeneous, and malignant tumours with calcification and necrosis [16,17]. Therefore, no single characteristic is fully reliable or conclusive, and understanding the limitations of CT is important when basing management strategies on radiological SRM evaluation.

Establishing reliable diagnostic criteria for malignant and benign SRM CT assessment could decrease the frequency of unnecessary interventions, which in turn would lower associated costs and risks. Therefore, the goals of this study were to evaluate which radiological tumour features were indicative of malignancy in cases without obvious aggressive growth or gross fat, and to demonstrate the diagnostic capabilities and limitations of MDCT scan imaging when it comes to determining the type and malignancy of incidentally detected lipid-poor SRMs.

## 2. Materials and Methods

### 2.1. Study Design, Setting, Population and Analysed Data

This was a retrospective single-centre study, performed in Lithuanian University of Health Sciences, Kaunas Clinics, Department of Radiology. We assessed medical data and MDCT imaging studies of 98 patients who underwent radiological and histopathological examination of incidentally diagnosed solid renal lesions between 1 January 2010 until 31 December 2016. Patients with histologically confirmed solid renal tumours (AML, CCC, papillary RCC (PRCC), chromophobe RCC (ChRCC), and oncocytoma) were included in the study if renal lesions were ≤4 cm in diameter or major axis length; were unexpectedly diagnosed during an abdominal US, further assessed by contrast enhanced MDCT; and in CT images, appeared without macroscopic fat or invasive growth. Patients with cystic lesions or masses less than 1 cm in diameter, insufficient medical record, inconclusive histological data, and poor quality or incomplete CT scans were excluded from the study.

Two certified abdominal radiologists with no knowledge of histological findings retrospectively assessed the MDCT scan images specifically to predict malignancy and histological types of renal tumours. Radiological conclusions were based on pre-determined and standardized criteria (intensity and homogeneity of radiographic contrast material accumulation, calcification, and central scarring), in addition to age and sex of the patient, and tumour localization (Table 1). All lesions were classified as either malignant or benign, and further sorted into histological categories: CCC, PRCC, ChRCC, AML, oncocytoma, or other benign lesion.

### 2.2. Scanning Parameters and Image Assessment

Four phase MDCT scan images were acquired using General Electric Light Speed Pro 64-layer CT machine with the patient in a supine position (arms raised above the head and legs straight). Subsequently to native image acquisition, patients received 100 mL of non-ionic intravenous contrast agent at the injection rate of 3.5 mL/s. Arterial, venous, and excretory phase images were obtained 25, 100, and 300 s following contrast agent injection. Standard scanning protocol was utilized in all cases: images were acquired using 5 mm thick slices, tube voltage of 120 kV, current of 80–750 mA, rotation speed of 0.8 s, and pitch of 1.375:1. Picture Archiving and Communication System (PACS) and multi-planar reconstructions (MPRs) were utilized during the assessment of CT scan images.

### 2.3. Statistical Analysis

Statistical analysis was performed using SPSS for Windows 20.0 and Microsoft Excel 5.00. Differences between groups were assessed using Mann–Whitney U and Chi-square statistical tests. To assess the significance of SRM characteristics (diameter, affected third, growth, scarring, and enhancement) when predicting the malignancy of a lesion on MDCT, univariate and multivariate logistic regressions were performed. SRMs were considered exophytic if more than 50% of the tumour was outside renal parenchyma, while lesions with less than 50% of their mass outside the parenchyma were considered endophytic. Multivariate logistic regression analysis included parameters that were *p* < 0.1 in univariate analysis: localisation (superior, middle, or inferior third), homogeneity of contrast agent accumulation, scarring, and the diameter of the tumour. Histopathological findings were interpreted as an indisputable diagnosis and compared to radiologists’ conclusions. When diagnoses of (non-)malignancy and/or type coincided, imaging conclusions were determined as correct. Additionally, correct prediction of only malignant or benign lesions were analysed. The sensitivity (SE) and specificity (SP) of a MDCT scan when differentiating between SRMs was determined by comparing the radiological diagnosis of malignancy and lesion type to documented conclusions of a pathologist.

Normally distributed data were expressed as the mean value with 95% confidence intervals (CI), with non-normally distributed data as the median value (quartiles). Values of *p* less than 0.05 were considered significant.

## 3. Results

The study population consisted of 98 (57 female and 41 male) patients with the median age of 66 (58–74) years; 63 (64%) patients were diagnosed with malignant and 35 (36%) with benign tumours; 49% of all tumours were CCCs, 22% oncocytomas, 12% AMLs, 9% PRCCs, 6% ChCCs, and 2% were other types of benign SRMs. The age and sex of study participants, and the differences in localisation and growth of SRMs between the two groups were not statistically significant. (Table 2).

**Figure 1:**
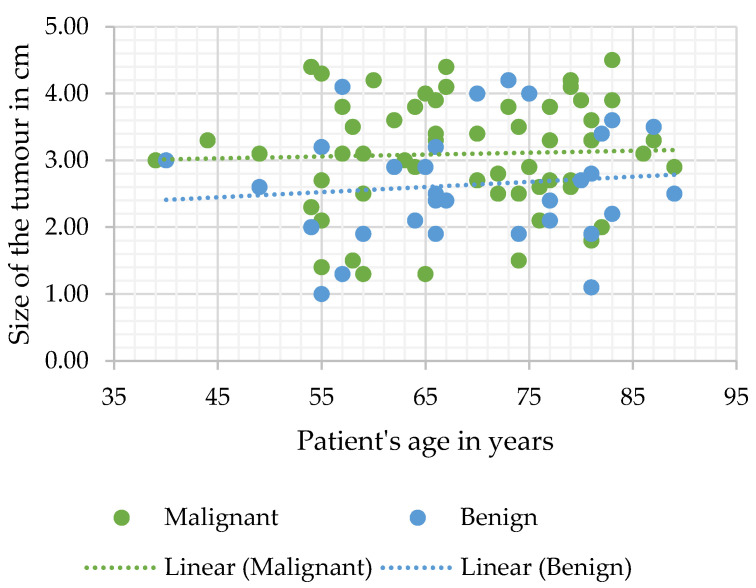
Tumour diameter measurement distribution according to age.

Heterogenous contrast agent accumulation and central scarring were more common in the malignant tumour group; however, this difference was statistically insignificant. Malignant tumours were statistically significantly bigger in diameter than benign SRMs (median measurement was 3.1 (2.6–3.8) cm versus 2.5 (2–3.2) cm, respectively, *p* = 0.006) (Figure 1).

Additionally, the diameter of SRMs proved to be significant when determining the correct diagnosis in both the univariate and multivariate logistic regression analysis of all study cases (*n* = 98) (OR 1.76, 95% CI 1.2–2.5, and *p* = 0.003; and OR 2.23, 95% CI 1.39–3.56, and *p* = 0.001 accordingly).

In malignant tumour group (*n* = 63), the univariate logistic regression analysis demonstrated that all included parameters were significant when determining the correct diagnosis. However, in multivariate analysis of the same group, only the diameter, homogenous enhancement, and central scarring were observed to be statistically significant (*p* = 0.003, *p* = 0.03, and *p* = 0.001, respectively (Table 3)).

Meanwhile, both univariate and multivariate analysis of benign masses (*n* = 35) determined that evaluating whether the growth was endophytic or exophytic was the only significant characteristic that increased the accuracy of the diagnosis (odds ratio (OR) 3.2, 95% CI (1.02–10.01), and *p* = 0.05; and OR 3.8, 95% CI (1.12–12.84), and *p* = 0.03, respectively). Other parameters included in this analysis were the affected third of the kidney and heterogeneous/homogeneous accumulation of contrast agent, however both were statistically insignificant for increasing the accuracy of benign lesion prediction.

In this study, we observed that the most sensitive predictive parameter of SRM malignancy or indolence was the presence of central scarring. However, it had low specificity (SE 91.3; SP 22.9) and, therefore, proved to be less diagnostic than diameter (SE 52.4, SP 74.3) and homogeneity of enhancement (heterogeneous accumulation SE 62.7; SP 51.4, homogeneous—SE 20.6; SP 68.6) (Table 4 and Table 5).

**Figure 2:**
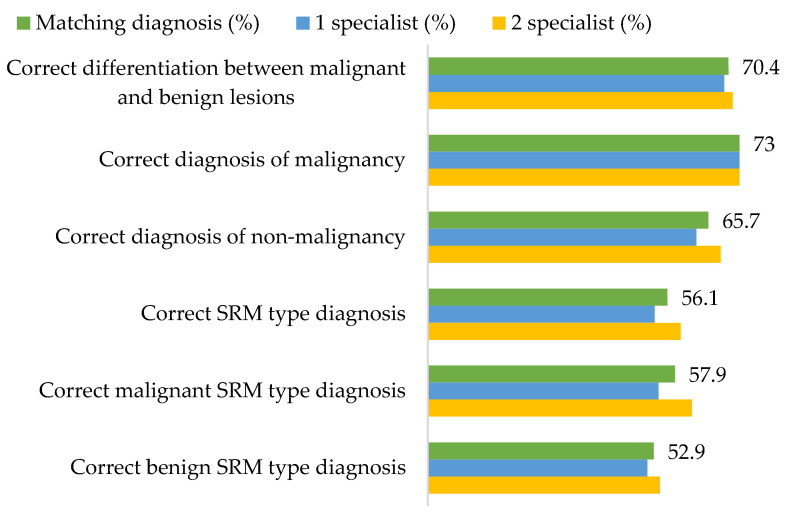
The accuracy of radiological small renal mass (SRM) type diagnosis and differentiation between malignant and benign SRMs.

The two abdominal radiologists, who evaluated MDCT scan images using standardized diagnostic criteria, agreed on malignancy or lack thereof in 68 cases, and the overall correct choice for the diagnosis was 70.4%. While the radiological conclusion of SRM type matched histopathological findings in 56.1% of cases, 73% of malignant lesions were identified as such (Figure 2).

## 4. Discussion

Radiological imaging plays a crucial role in renal tumour detection, assessment, and management planning. However, in clinical practice, it is currently believed that CT/Magnetic resonance imaging (MRI) and other imaging modalities do not reliably determine the malignancy of incidentally detected SRMs, and biopsy or surgical treatment is required for most masses without gross fat [9]. This means that with growing numbers of incidentally detected SRMs, the amount of surgical and ablative therapy treatments has increased as well. Nonetheless, RCC mortality has not declined [2,18]. Multiple factors are undoubtedly responsible for such tendencies; however, it is likely related to the fact that, according to various publications and our own study, approximately 30% of SRMs may be benign, and even malignant tumours are not equally aggressive [19,20,21,22,23,24,25]. For this reason, current management strategies should be reviewed, and less invasive renal lesion evaluation methods, namely radiological imaging, should be investigated.

**Figure 3:**
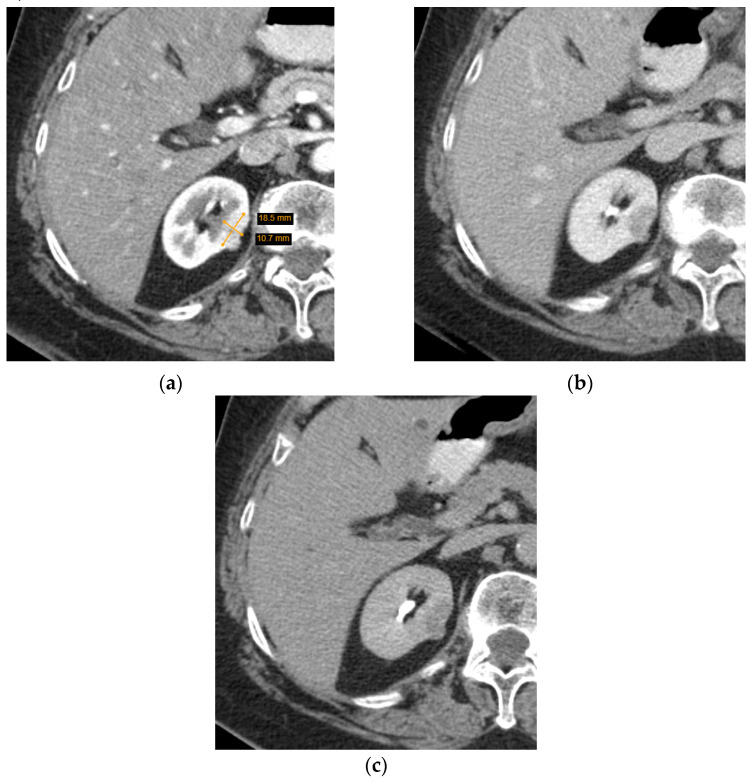
Axial computed tomography (CT) scan images of a small heterogenous enhancing renal tumour. The lesion was radiologically indicative of a renal cell carcinoma, while histological diagnosis was oncocytoma: (**a**) arterial phase; (**b**) portovenous phase; (**c**) excretory phase.

**Figure 4:**
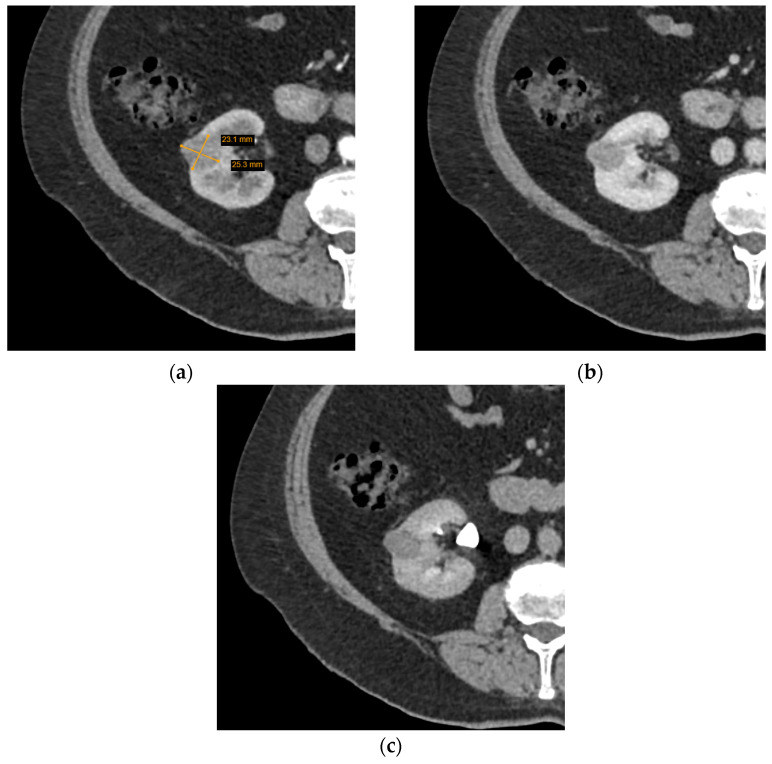
Axial computed tomography (CT) scan images of a small homogeneous enhancing renal tumour. The lesion was radiologically indicative of oncocytoma, while histological diagnosis was chromophobe cell carcinoma: (**a**) arterial phase; (**b**) portovenous phase; (**c**) excretory phase.

CT is more commonly utilized for SRM assessment than MRI and has greatly improved with the invention of MDCT—a method of CT that yields higher resolution images, decreases frequency of artifacts, and improves MPR image quality. Several characteristics of a renal lesion in four-phase MDCT are helpful: macroscopic fat is commonly regarded as a characteristic feature of AMLs [26]; bigger lesions are more likely to be malignant [24,25], while central scarring and certain enhancement characteristics are indicative of both oncocytoma and different types of RCCs [27,28]. Unfortunately, lipid-poor SRMs are rather difficult to evaluate; scarring, calcification, and enhancement homogeneity may be observed in different tumours or difficult to distinguish due to the small size, and no feature by itself can determine the malignancy, let alone type of the SRM. Herein lies the dilemma—if no feature is diagnostic and lipid-poor SRMs may manifest atypically, is it reasonable to guide management based on MDCT conclusions (Figure 3 and Figure 4)?

In our study, we assessed the subpopulation of patients with incidentally detected lipid-poor SRMs that underwent surgical treatment due to initially inconclusive or doubtful radiological diagnosis. We noticed that some features of SRMs were more indicative of malignancy than others, and affected the accuracy of radiologist’s conclusions. First of all, we concluded that there is a direct correlation between the size of the tumour and the likelihood of malignancy (Figure 1). These findings were unsurprising, seeing as multiple studies have observed the same relationship between the size of the lesion and the likelihood of malignancy [29]. Admittedly, sensitivity was moderate (SE 52.4), but it was the most specific parameter (SP 74.3), and evaluating the diameter of the mass significantly influenced the accuracy of CT diagnosis. Heterogenous contrast accumulation, meanwhile, which was more sensitive and less specific (SE 62.7 and SP 51.4) than the diameter of the tumour, proved to be significant in the univariate logistic analysis of the malignant tumour group and, while insignificant in multivariate analysis of the same group, was more commonly observed in malignant SRMs. These conclusions are in agreement with other studies, suggesting that heterogenous large tumours are more likely to be malignant, and evaluating these parameters improves the reliability of the radiologist’s diagnosis. Although further research is still required, advances in machine-learning such as texture analysis, an objective evaluation of tissue density that discerns subtle changes in lesion texture, have further improved evaluation of tumour enhancement patterns and the reliability of CT scan conclusions [30,31,32]. Additionally, we determined that central scarring significantly influenced the accuracy of the diagnosis and was very sensitive, however, unspecific (SE 91.3; SP 22.9). This is also in agreement with the current understanding that scarring of an oncocytoma cannot always be differentiated from necrosis in RCC and may be observed only in so little as a third of all cases [33,34,35]. All of the MDCT SRM parameters evaluated in our study were non-specific and require further research before optimal assessment criteria for malignancy and SRM types can be established.

As stated above, none of the SRM features are diagnostic. Therefore, identifying the malignancy of the lesion and its type in MDCT scan images is a challenge. We observed that in 70.4% of cases, the radiological conclusion whether the lesion was malignant or benign was correct, and benign lesions were more commonly diagnosed as malignant (34.3% of cases) than vice versa. Other studies have also observed that MDCT can successfully differentiate both lipid-poor AMLs from RCCs, and benign lesions from malignant ones, although further improvement is required [15,17,36,37,38]. Unfortunately, while MDCT can relatively adequately determine lesion malignancy, when it comes to predicting the histological type of the SRM, histological analysis remains superior. In our study, it was accurately predicted in only 56.1% of the cases, leading to the conclusion that MDCT determined SRM type was not reliable.

Unsurprisingly, MDCT is not a fool-proof diagnostic tool and has shown to be rather unreliable when determining the histological type of the lesion, or misidentifying malignant SRMs as benign and vice versa, but what does that mean for patient survival and management strategies? A large proportion of patients with incidentally detected SRMs are older or may not be candidates for surgery. Non-invasive management is preferable in such cases, especially when the tumours are especially small, or the patients have multiple comorbidities that increase the likelihood of complications following intervention. In such instances, when the risks outweigh the possible benefits, or the mass is unlikely to be malignant, the patients may be placed under active surveillance [39]. Multiple publications, including prospective studies, have reported that active surveillance is safe in most cases, and does not decrease either overall or cancer-specific survival [40,41]. Therefore, even if the initial radiological diagnosis is incorrect, active observation would ensure that treatment, if necessary, would be provided in time. Nonetheless, improved MDCT evaluation algorithms would be beneficial both for pre-selecting patients for active surveillance and for detecting signs of malignancy during observation and, as such, should be optimized.

Although our results were mostly in accord with other studies, this study had some limitations. First of all, we assessed MDCT images of SRMs retrospectively in a single medical centre, and our selection criteria excluded patients with macroscopic fat or clear signs of malignancy. While this permitted us to evaluate the benefits of MDCT imaging in typically inconclusive lesions, and our findings were in accordance to other studies, further prospective research with inclusion of all SRMs is required to validate the credibility of our conclusions. Secondly, we assessed the accuracy of radiological diagnoses by assuming that histopathological conclusions were indisputable and true. It is important to note that even pathological analysis has its own pitfalls. Nonetheless, we believe that it should not have influenced the results. Tissue samples were provided following resection or nephrectomy, and currently, it is the most accurate method of determining malignancy and type of the lesion. Finally, some lesions included in the study had exceeded the 4 cm cut-off value. The reason for this aberration in patient selection was due to the different measurements of SRMs; while one radiologist had evaluated the lesion as ≤4 cm in diameter, the second radiologist had measured it >4 cm. However, the conclusions of our study did not differ from those in other recent studies, and it is unlikely that inclusion of the aforementioned patients affected the results.

## 5. Conclusions

To conclude, in the setting of incidentally identified lipid-poor SRMs without clear signs of aggressive growth, MDCT four-phase scan evaluation by an experienced abdominal radiologist can determine the malignancy of the lesion with adequate accuracy. Central scarring, endophytic/exophytic growth, and contrast agent accumulation homogeneity must always be taken into consideration when determining whether the SRM in question is malignant or not, yet SRM diameter is the most reliable parameter and is directly proportional to the possibility of malignancy. Therefore, although MDCT cannot accurately predict the histopathological SRM type, in certain cases, specifically when surgical treatment and invasive diagnostic procedures are discouraged or unavailable, it is reasonable to base management choices on radiological conclusions.

## Figures and Tables

**Table 1:** Computed tomography characteristics of individual solid renal masses.

Tumour	Accumulation of Contrast Agent			Calcification	Central Scarring
	Homogeneous	Heterogeneous	Poor		
CCC *	−	+	−	+	+/−
PRCC *	−	−	+	+	−
ChRCC *	−	−	+	+/−	+
AML *	+	−	−	−	−
Oncocytoma	+	−	−	−	+

* CCC—clear cell carcinoma; PRCC—papillary renal cell carcinoma; ChRCC—chromophobe renal cell carcinoma; AML—angiomyolipoma.

**Table 2:** The frequency of malignant and benign small renal masses (SRMs) depending on the sex, localization, and growth of the mass.

Parameters	Malignant Tumours *n* = 63	Benign Tumours *n* = 35	*p*	All Tumours *n* = 98
Sex (%)				
Female	37 (58.7)	20 (57.1)	0.88	57 (58.2)
Male	26 (41.3)	15 (42.9)		41 (41.8)
Affected kidney, *n* (%)				
Right	28 (44.4)	13 (57.1)	0.53	41 (41.8)
Left	35 (55.6)	22 (42.9)		57 (58.2)
Diameter of the tumour (cm), median (quartiles)	3.1 (2.6–3.8)	2.5 (2–3.2)	0.006	2.9 (2.4–3.6)
Age (years), median (quartiles)	66 (57–72)	66 (59–80)	0.16	66 (58–74)
Localisation of the tumour, *n* (%)				
Superior third	14 (22.2)	8 (22.9)		22 (22.4)
Middle third	24 (38.1)	15 (42.9)	0.9	39 (39.8)
Inferior third	25 (39.7)	12 (34.3)		37 (37.8)
Localisation of the tumour, *n* (%)				
Peripheral part	56 (88.9)	32 (91.4)	0.9	88 (98.8)
Central part	7 (11.1)	3 (8.6)		10 (10.2)
Growth of the tumour, *n* (%)				
Exophytic	20 (31.7)	13 (37.1)	0.65	33 (33.7)
Endophytic	43 (68.3)	22 (62.9)		65 (66.3)
Contact with renal pelvis/calyces, *n* (%)				
No	32 (50.8)	24 (68.6)	0.13	56 (57.1)
Yes	31 (49.2)	11 (31.4)		42 (42.9)

**Table 3:** Univariate and multivariate analysis of parameters influencing the diagnostic accuracy of computed tomography (CT) in the malignant tumour group (*n* = 63).

	Univariate Analysis			Multivariate Analysis		
Parameters	OR *	95% CI *	*p*	OR *	95% CI *	*p*
Diameter of the tumour	2.1	(1.29–3.54)	0.003	2.4	(1.36–4.18)	0.003
Middle third of the kidney	2.8	(1.23–6.17)	0.01	1.8	(0.69–4.61)	0.2
Exophytic growth	3.0	(1.32–6.82)	0.009	0.5	(0.18–1.34)	0.2
Heterogeneous accumulation of c/a **	4.1	(1.79–9.38)	0.001	0.8	(0.25–2.85)	0.8
Homogeneous accumulation of c/a **	5.8	(2.3–14.66)	0.001	3.4	(1.13–9.90)	0.03
Central scar	16.2	(3.29–79.84)	0.001	10.6	(1.75–64.60)	0.001

* OR—odds ratio, CI—confidence interval, ** c/a—contrast agent.

**Table 4:** The sensitivity, specificity, positive predictive value (PPV) and negative predictive value (NPV) of parameters influencing the diagnostic accuracy of computed tomography (CT).

Parameter	Sensitivity	Specificity	PPV	NPV
Heterogeneous accumulation of c/a *	62.7	51.4	69.9	43.4
Homogeneous accumulation of c/a *	20.6	68.6	54.2	32.4
Central scar	91.3	22.9	68.0	59.3
Exophytic growth	68.3	37.1	66.2	39.4
Middle third	38.1	57.1	61.5	33.9
The diameter of the tumour ≤ 3 vs. >3 cm	52.4	74.3	78.6	46.4

* c/a—contrast agent.

**Table 5:** Accumulation of contrast agent (c/a), presence of calcification, and central scarring in malignant and benign tumours.

Parameters	Malignant Tumours, *n* = 63	Benign Tumours, *n* = 35	*p*	All Tumours, *n* = 98
Accumulation of c/a, *n* (%)				
Poor	12 (19.0)	7 (20.0)		19 (19.4)
Homogeneous	13 (20.6)	11 (31.4)	0.44	24 (24.5)
Heterogeneous	38 (60.3)	17 (48.6)		55 (56.1)
Calcification, *n* (%)				
Yes	57 (90.5)	33 (94.3)	0.7	90 (91.8)
No	6 (9.5)	2 (5.7)		8 (8.2)
Central scarring, *n* (%)				
Yes	55 (87.3)	25 (71.4)	0.06	80 (81.6)
No	8 (12.7)	10 (28.6)		18 (18.4)

## Data Availability

Data available on request from the authors.
